# Involvement of homodomain interacting protein kinase 2‐c‐Jun N‐terminal kinase/c‐Jun cascade in the long‐term synaptic toxicity and cognition impairment induced by neonatal Sevoflurane exposure

**DOI:** 10.1111/jnc.14910

**Published:** 2020-01-19

**Authors:** Lirong Liang, Rougang Xie, Rui Lu, Ruixue Ma, Xiaoxia Wang, Fengjuan Wang, Bing Liu, Shengxi Wu, Yazhou Wang, Hui Zhang

**Affiliations:** ^1^ State Key Laboratory of Military Stomatology & National Clinical Research Center for Oral Diseases & Shaanxi Engineering Research Center for Dental Materials and Advanced Manufacture, Department of Anethesiology School of Stomatology Fourth Military Medical University Xi’an Shaanxi P. R. China; ^2^ Department of Neurobiology and Institute of Neurosciences School of Basic Medicine Fourth Military Medical University Xi’an Shaanxi P. R. China

**Keywords:** cognition, homeodomain interacting protein kinase 2, sevoflurane, synaptic toxicity

## Abstract

Sevoflurane is one of the most widely used anesthetics with recent concerns rising about its pediatric application. The synaptic toxicity and mechanisms underlying its long‐term cognition impairment remain unclear. In this study, we investigated the expression and roles of homeodomain interacting protein kinase 2 (HIPK2), a stress activating kinase involved in neuronal survival and synaptic plasticity, and its downstream c‐Jun N‐terminal kinase (JNK)/c‐Jun signaling in the long‐term toxicity of neonatal Sevoflurane exposure. Our data showed that neonatal Sevoflurane exposure results in impairment of memory, enhancement of anxiety, less number of excitatory synapses and lower levels of synaptic proteins in the hippocampus of adult rats without significant changes of hippocampal neuron numbers. Up‐regulation of HIPK2 and JNK/c‐Jun was observed in hippocampal granular neurons shortly after Sevoflurane exposure and persisted to adult. 5**‐**((6‐Oxo‐5‐(6‐(piperazin‐1‐yl)pyridin‐3‐yl)‐1,6‐dihydropyridin‐3‐yl)methylene)thiazolidine‐2,4‐dione trifluoroacetate, antagonist of HIPK2, could significantly rescue the cognition impairment, decrease in long‐term potentiation, reduction in spine density and activation of JNK/c‐Jun induced by Sevoflurane. JNK antagonist SP600125 partially restored synapse development and cognitive function without affecting the expression of HIPK2. These data, in together, revealed a novel role of HIPK2‐JNK/c‐Jun signaling in the long‐term synaptic toxicity and cognition impairment of neonatal Sevoflurane exposure, indicating HIPK2‐JNK/c‐Jun cascade as a potential target for reducing the synaptic toxicity of Sevoflurane.

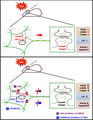

Cover Image for this issue: doi: 10.1111/jnc.14757.

## Graphical abstract

Sevoflurane is one of the most widely used pediatric anesthetics. Its long‐term effects on synapse development and cognition remain poorly understood. Here, we report that neonatal Sevoflurane exposure results in impairment of memory, enhancement of anxiety, reduction in excitatory synapses in the adult hippocampus with up‐regulation of HIPK2 and JNK/c‐Jun signaling. A64, antagonist of HIPK2, could significantly rescue the cognition impairment, synaptic dysfunction, and JNK/c‐Jun activation. JNK antagonist SP600125 partially restored synapse development and cognitive function without affecting HIPK2. These data revealed a novel role of HIPK2‐JNK/c‐Jun cascade in the long‐term synaptic toxicity of neonatal Sevoflurane exposure.

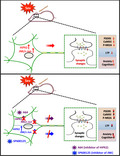

**Abbreviations used**
A64: 5‐((6‐Oxo‐5‐(6‐(piperazin‐1‐yl)pyridin‐3‐yl)‐1,6‐dihydropyridin‐3‐yl)methylene)thiazolidine‐2,4‐dione trifluoroacetate
ACC: anterior cingulate cortex
ALS: amyotrophic lateral sclerosis
CaMKII: calcium/calmodulin‐dependent kinase II
CC3: cleaved caspase 3
CNS: Central nervous system
CPu: caudate putamen
ER: endoplasmic reticulum
HIP: hippocampus
HIPK2: homodomain interacting protein kinase 2
JNK: c‐Jun N‐terminal kinase
NR2A: *N*‐methyl‐d‐aspartate receptor subunit 2 A
PSD95: post‐synaptic density protein 95
Sev: sevoflurane
vGAT: vesicular inhibitory amino acid transporter

Millions of babies are subjected to general anesthesia every year. Because of the high sensitivity of neonatal central nerve system (CNS) to anesthetics, the safe use of general anesthetics in children has been held as an important issue both by doctors and by publics. Recently, animal studies in rodents and primates have demonstrated that long‐term or repeated exposure to anesthetics in neonatal could exert irreversible effects on the development of central nerve system (CNS) (Amrock *et al*. 2015; Long Ii *et al.* 2016; Raper *et al.* 2018; O'Farrell *et al.* 2018; Li *et al.* 2019), possibly resulting decrease in learning ability, abnormal social behavior, and impairment of cognition. Revealing the underlying mechanism is of great value for reducing the potential neurotoxicity of neonatal anesthesia.

Sevoflurane (Sev) is one of the most widely used pediatric anesthetics (Lerman *et al.* 1994; Yeo *et al.* 2007; Costi *et al.* 2014). It has been reported that early exposure to Sev may lead to long‐term behavioral changes and cognitive abnormalities later on in life in rodents (Ji *et al.* 2017; Xia *et al.* 2017; Tang *et al.* 2018; Yu *et al.* 2018) and in non‐human primates (Liu *et al.* 2015; Raper *et al.* 2015; Alvarado *et al.* 2017). The neurotoxicity of Sev has been mainly attributed to its apoptosis‐inducing effects (Satomoto *et al.* 2009; Takaenoki *et al.* 2014; Ozer *et al.* 2017; Bi *et al.* 2018; Xie and Wang 2018). Considering that Sev exposure affects the efficiency of synapse transmission (Naruo *et al.* 2005; Haseneder *et al.* 2009; Kotani and Akaike 2013) and that synaptogenesis occurs mainly in postnatal stage, it is possible that Sev exposure may influence the development of synapse and subsequently lead to cognition and memory impairment, which remains poorly investigated.

Homodomain interacting protein kinase 2 (HIPK2) is a member of an evolutionary conserved family of serine/threonine kinases. It is generally considered as a tumor suppressor (Saul and Schmitz 2013; Conrad *et al.* 2016; Choi *et al.* 2017; Feng *et al.* 2017). In nervous system, HIPK2 is expressed from late embryonic stage and has been demonstrated to play important roles in the neuronal survival and degeneration both in normal development and under pathological conditions, such as amyotrophic lateral sclerosis (Lee *et al.* 2016; Conte and Pierantoni 2018). Whether HIPK2 and its downstream signals respond to Sev exposure and involved in the process of synaptic degeneration remains unknown.

In this study, we explored the long‐term effects of neonatal Sev exposure on memory and anxiety behaviors. Our data revealed a role of HIPK2 and its downstream c‐Jun N‐terminal kinase (JNK) signaling in this process.

## Materials and methods

### Animals

All the SD male rats (neonatal and adult, RRID:RGD_1566457) were bought from the animal center of the Fourth Military Medical University. All animal experiments were carried out under protocols approved by the Animal Care and Use Committee of Fourth Military Medical University (ethical approval reference number: No. 077 of 2017) and according to ‘Policies on the Use of Animals and Humans in Neuroscience Research’ revised and approved by the Society for Neuroscience in 1995. 258 rats (eight rats per group for behavior assays and six rats per group for other experiments) in total were used in this study. The study was not pre‐registered. No randomization was performed to allocate subjects in the study.

### Experimental design and Sevoflurane treatment

Neonatal SD rats at P6 were assigned to the following groups: control group, Sev group (Cat #: H20110142), Sev plus 5**‐**((6‐Oxo‐5‐(6‐(piperazin‐1‐yl)pyridin‐3‐yl)‐1,6‐dihydropyridin‐3‐yl)methylene)thiazolidine‐2,4‐dione trifluoroacetate (A64) (inhibitor of HIPK2; Sigma‐Aldrich, St. Louis, MO, USA, SML 1731) group, and Sev plus SP600125 (inhibitor of JNK; Sigma‐Aldrich, S5567) group, A64 group, SP600125 group. In Sev group, rats were treated with 3% Sev plus 60% oxygen (balanced with nitrogen) for 2 h/day for three consecutive days. In control group, rats were treated with 60% oxygen (balanced with nitrogen) for 2 h/day for three consecutive days (Shen *et al.* 2013; Lu *et al.* 2017). In Sev plus A64 group, rats were treated with A64 (100 mg/kg) at 1 h before anesthesia each day. In Sev plus SP600125 group, rats were treated with SP600125 (10 mg/kg) at 1 h before anesthesia each day. In Sev group, saline or DMSO were injected i.p. 1 h before anesthesia as control. All treatments were carried out at 8:00–10:00 AM of the day. When performing intraperitoneal injection of A64 and SP600125, rats were carefully handled and small size of needles was used to reduce pain. Free water and food were available for animals during all the procedure of experiments. Arterial blood gas analysis has been performed at the end of anesthesia to ensure that there was no hypoxia. To prevent hypothermia, a warming blanket was used during anesthesia. The effects of Sev on the behavior and synapse development were first assessed. Then, the effects of A64 were evaluated. In the end, the effects of SP600125 were investigated.

### Immunohistochemistry

For immunohistochemistry, rats were anesthetized by using 4% chloral hydrate and then perfused intracardially with 4% paraformaldehyde phosphate buffer. Serial coronal sections were prepared. Slides were blocked by phosphate‐buffered saline (PBS) containing 3% bovine serum albumin and 0.3% Triton‐X100, and then incubated with primary antibodies overnight at 25℃ as the following: rabbit anti‐ HIPK2 antibody (Abcam, 1 : 200, Cambridge, MA, USA, RRID:AB_732895), rabbit anti‐c‐Jun antibody (Abcam, 1 : 200, Cat# ab40766), rabbit anti‐cleaved caspase 3 (CC3) antibody (Genetex, Irvine, CA, USA, 1 : 100, Cat# GTX22302)，rat anti‐NeuN antibody (Abcam, 1 : 600, Cat#ab104224), rat anti‐GFAP antibody (Abcam, 1 : 300, Cat# ab10062), goat anti‐Iba1 antibody (Thermo Fisher Scientific, Waltham, MA, USA, 1 : 100, Cat#PA5‐18039), and goat anti‐CC1 antibody (Santa Cruz Biotechnology, Santa Cruz, CA, USA, 1 : 200, Cat# ab16794). After washing with PBS, corresponding secondary antibodies conjugated with Alexa Fluro 488 (donkey anti‐rabbit; Jackson Immuno‐Research, West Grove, PA, USA, 1 : 500, RRID:AB_2313584) or Alexa Fluro 594 (donkey anti‐goat, donkey Anti‐mouse; Jackson ImmunoResearch, West Grove, PA, USA, 1 : 500, RRID:AB_2340433, RRID:AB_2340854) were incubated with the sections for 2–4 h at 25℃ protected from light. After washing with PBS, sections were counter‐stained with Hoechst33324 (1 : 1000; Sigma, St. Louis, MO, USA) for 20 min.

### Golgi staining

Rats were perfused with PBS. Brains were immersed in Golgi staining solution which contained 50% potassium dichromate (MP 021563389), 5% Mercuric chloride (Sigma‐Aldrich, M1136), and 5% Potassium chromate (Sigma‐Aldrich, 529508) and protected from light. Brain sections with thickness of 150 μm were made at 7 days after incubation. When performing Golgi staining, sections were washed with distilled water, dehydrated with ethanol, and then treated with ammonia (3 : 1). The sections were subsequently washed by distilled water and incubated in 5% sodium thiosulfate for 10 min, and then dehydrated with degraded ethanol and clarified with xylene. Then, the sections were observed under bright field of Olympus FV1000 (Tokyo, Japan). The images were taken by z‐stack scanning with the excitation wavelength of 405 nm, and the virtual color was converted into green color. For spine density and subtype analysis, dendrites were reconstructed by using IMARIS and the spines were grouped according to the following criteria as described (Kang *et al.* 2017). Tubby: length (spine) < 1.5 and max width (head) < mean_width (neck) *1.2; mushroom: max width (head) > mean width (neck) *1.2 and max_width (head) > 0.3; if the spine was not classified as mushroom or stubby, it was defined as long‐thin.

### Electron microscopic study

Animals were perfusion fixed with a mixture of 4% paraformaldehyde containing 1% glutaraldehyde. Tissue sections of 50 μm were prepared with a vibratome and further fixed with 1% osmium tetroxide, dehydrated with graded ethanol, replaced with propylene oxide, and flat‐embedded in Epon 812. The sections were trimmed under a stereomicroscope and mounted onto blank resin stubs for ultrathin sectioning. Ultrathin sections (70–80 nm) were prepared on an LKB Nova Ultratome (LKB, Bromma, Sweden). After being counter‐stained with uranyl acetate and lead citrate, the sections were examined under a JEM‐1230 electron microscope (JEM, Tokyo, Japan).

### Electrophysiological recording

Rats were killed under anesthesia by chloral hydrate. Then, the hippocampi were isolated for sectioning into 400‐μm‐thick slices in ice‐cold saline using a vibratome (VS1200; Leica, Solms, Germany). The slices were maintained at 25℃ in an interface holding chamber saturated with 95% O_2_ and 5% CO_2_. After recovery for at least 1 h, the slices were transferred into the recording chamber maintained at 30°C.

For extracellular recording of field post‐synaptic potentials (fPSPs), the pipettes were filled with ACSF and placed in the stratum radiatum of dorsal CA1. The Schaffer collateral pathway was stimulated with 0.1 ms pulses delivered by a concentric bipolar stimulating electrode (FHC, Inc.; Bowdoin, ME, USA). To record the plasticity of fPSPs, the slope of fPSPs was measured at baseline for at least 10 min with a stimulation frequency of 0.05 Hz and using a stimulation intensity that produced a half‐maximal response. Slices were then stimulated at 100 Hz 1 s with 1 s interval for four times, and fPSPs were monitored for 60 min after stimulation.

### Western‐blotting

Hippocampal tissues were homogenized in RIPA lysis buffer containing proteinase inhibitor. Protein concentration was measured by BCA assay. Protein samples were separated by 10–15% gel. After SDS‐PAGE and protein was transferred to PVDF membrane. Membranes were blocked with TBS containing 5% non‐fat milk and 0.1% Tween 20 for 1 h at 25℃) and then incubated with primary antibodies overnight at 4°C as the following: rabbit anti‐HIPK2 antibody (Fitzgerald, North Acton, MA, USA, 1 : 3000, Cat#:70R‐50955), rat anti‐β‐actin antibody (Sigma‐Aldrich, 1 : 10000, Cat#:A5441)，rabbit anti‐cleaved‐caspase 3 antibody (Cell Signaling Technology, Beverly, MA, USA, 1 : 500, Cat#:9664), rabbit anti‐Synaptophysin antibody (Abcam, 1 : 1000, Cat#: ab8049), rat anti‐post‐synaptic density protein 95 (PSD95) antibody (Abcam, 1 : 1000, Cat#:ab18258), rabbit anti‐calcium/calmodulin‐dependent kinase II (CaMKII) antibody (Abcam, 1 : 1000, Cat#:ab52476), rabbit anti‐NMDAR2A antibody (phospho Y1325) (Abcam, 1 : 1000, Cat#:ab16646), rabbit anti‐NMDAR2A antibody（(Abcam, 1 : 1000, Cat#:ab124913), rabbit anti‐vesicular inhibitory amino acid transporter (vGAT) antibody (Abcam, 1 : 500, Cat#:ab42939), rabbit anti‐JNK1/2/3 (Abcam, 1 : 1000, Cat#:ab179461), rabbit anti‐JNK1/2/3 (phospho T183 + T183+T221) (Abcam, 1 : 1000, Cat#:ab124956), rabbit anti‐ c‐Jun antibody (Abcam, 1 : 1000, Cat#:ab40766). After washing with TBST, membranes were incubated with incubation with HRP‐conjugated anti‐rabbit or anti‐rat IgG (1 : 5000; Proteintech, Wu Han, China, Cat#:SA00001‐2, Cat#:SA00001‐15) for 1 h at 25℃. Bands were visualized with an ECL kit (Thermo, Waltham, MA, USA, Cat#:32106). Images were analyzed by Image J Software National Institutes of Health (NIH, Bethesda, MA, USA).

### Morris water maze test

Water maze was made by a round container with the diameter of 180 cm and the wall of 70 cm. The water maze was filled with water (25°C) and divided into four quadrants. The objective platform was placed 2 cm under water. For training, rats were put into water randomly in one quadrant facing the wall. The time when rats find the platform was recorded as escaping time (the rats which could not find the platform within 60 s were guided to the platform for learning). Rats were trained four times per day for 4 days. In the fifth day, rats were let swim freely in the maze. The total time rats stay in the in platform quadrant and the crossing times were recorded. The swimming traces and the escaping behavior were recorded using the software SMART 3.0.

### Conditioned fear memory assay

Rats were accustomed to experimental environment for 3 days before experiments. For the fear behavior test, rats were placed in fear box for 10 min in day 1. In day 2, rats were given five times of voice stimulation (20 s, 4 kHz, 80 dB) coupled with foot shocks (0.8 mA, 1 s). The first stimulation was given at 120 s after putting the animal into the fear box and 105 s intervals were set for each stimulation. After stimulation, rats were put back into home cage. In day 3, rats were placed in the fear box for 10 min. The animal behavior was recorded automatically. The percentages of rats exhibiting freezing was analyzed by a researcher blinded to experimental design.

### Open field test

Rats were accustomed to experimental environment for 5 days before experiments. The open field test was carried out in a black plastic chamber (100 × 100 × 50 cm) as described in the previous study (Huang *et al.* 2015) with slight modification. For each test, rat was gently placed in one corner, and the movement was recorded for 10 min with a video tracking system. The time spent and distance traveled in the central area, and the total distance traveled in the field were measured using the SMART software (SMART 3.0; Panlab S.L.U., Shenzhen, China).

### Elevated plus maze test

The elevated plus maze test was performed on the next day of the open‐field test. The maze was placed 50 cm above the floor and consisted of two open arms and two closed arms (50 × 10 cm and 30 cm wall height for the closed arms). Each rat was placed onto the center area, heading toward the same open arm, and videotaped in the following 5 min. The time spent and moving distance in the open arms, and the total movements in both open and closed arms were analyzed using the software SMART 3.0. The maze was cleaned by 75% ethanol between tests.

### Statistical analysis

**Figure 1:**
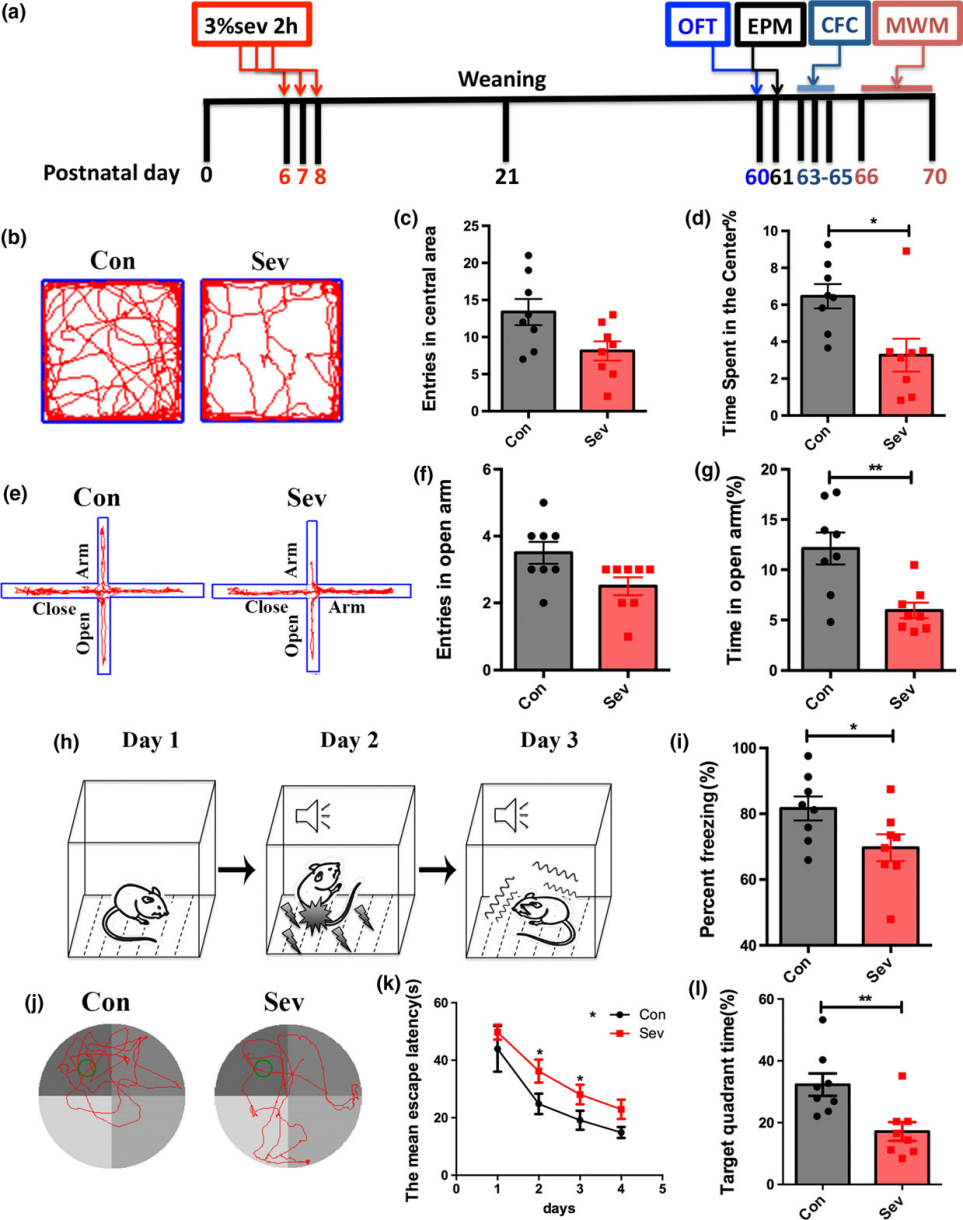
Effects of neonatal Sev exposure on anxiety and memory in adult. (a) Experimental design. (b–d) Open field assay of control rats and Sev‐treated rats. *N* = 8 rats per group. (e–g) Elevated plus maze test of control rats and Sev‐treated rats. *N* = 8 rats per group. (h, i) Conditioned fear memory assay. *N* = 8 rats per group. (j–l) Morris water maze test of control rats and Sev‐treated rats. *N* = 8 rats per group. Notice that neonatal repeated Sev exposure leaded to increase in anxiety and impairment of memory in adult. Con, control. Sev, Sevoflurane. *p < 0.05. **p < 0.01. Student’ *t*‐test (a–i). One‐way anova (j–l).

**Figure 2:**
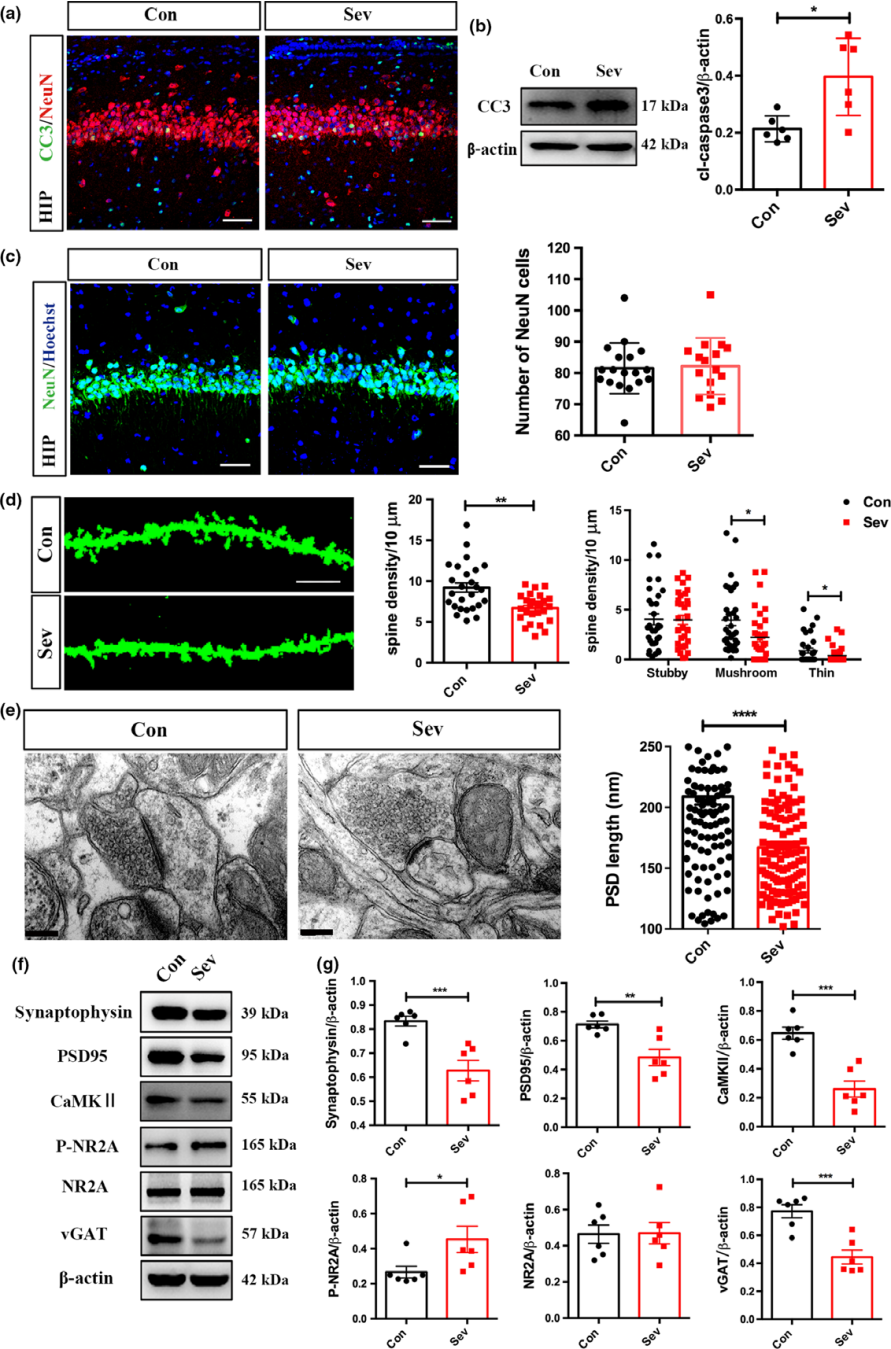
Effects of neonatal Sev exposure on granular neuron number and synapse development in adult hippocampus. (a, b) Double‐immunostaining of CC3/NeuN, and western blotting of CC3 in the hippocampus of control and Sev‐treated rats at 6 h post last Sev treatment. *N* = 6 rats per group. (c) Immunostaining and quantification of NeuN in the adult hippocampus of control rats and Sev‐treated rats. *N* = 6 rats per group. (d) Golgi staining and quantification of spines in control rats and Sev‐treated rats in adult. *N* = 6 rats per group. (e) Synaptic ultrastructure in the hippocampus of control and Sev‐treated rats in adult. *N* = 6 rats per group. (f, g) Western blotting and quantification of Synaptophysin, post‐synaptic density protein 95 (PSD95), calcium/calmodulin‐dependent kinase II (CaMKII), phosphorylation of *N*‐methyl‐d‐aspartate receptor subunit 2 A (pNR2A), NR2A, and vesicular inhibitory amino acid transporter (vGAT). *N* = 6 rats per group. Notice that Sev treatment significantly reduced the number of synapse as well as the expression of synaptic proteins. Con, control. Sev, Sevoflurane. *p < 0.05. **p < 0.01. ***p < 0.001. ****p < 0.0001. Student’ *t*‐test (b, c, e–g). One‐way anova (d). Bar = 50 μm (a–c), 10 μm (d), 200 nm (e).

**Figure 3:**
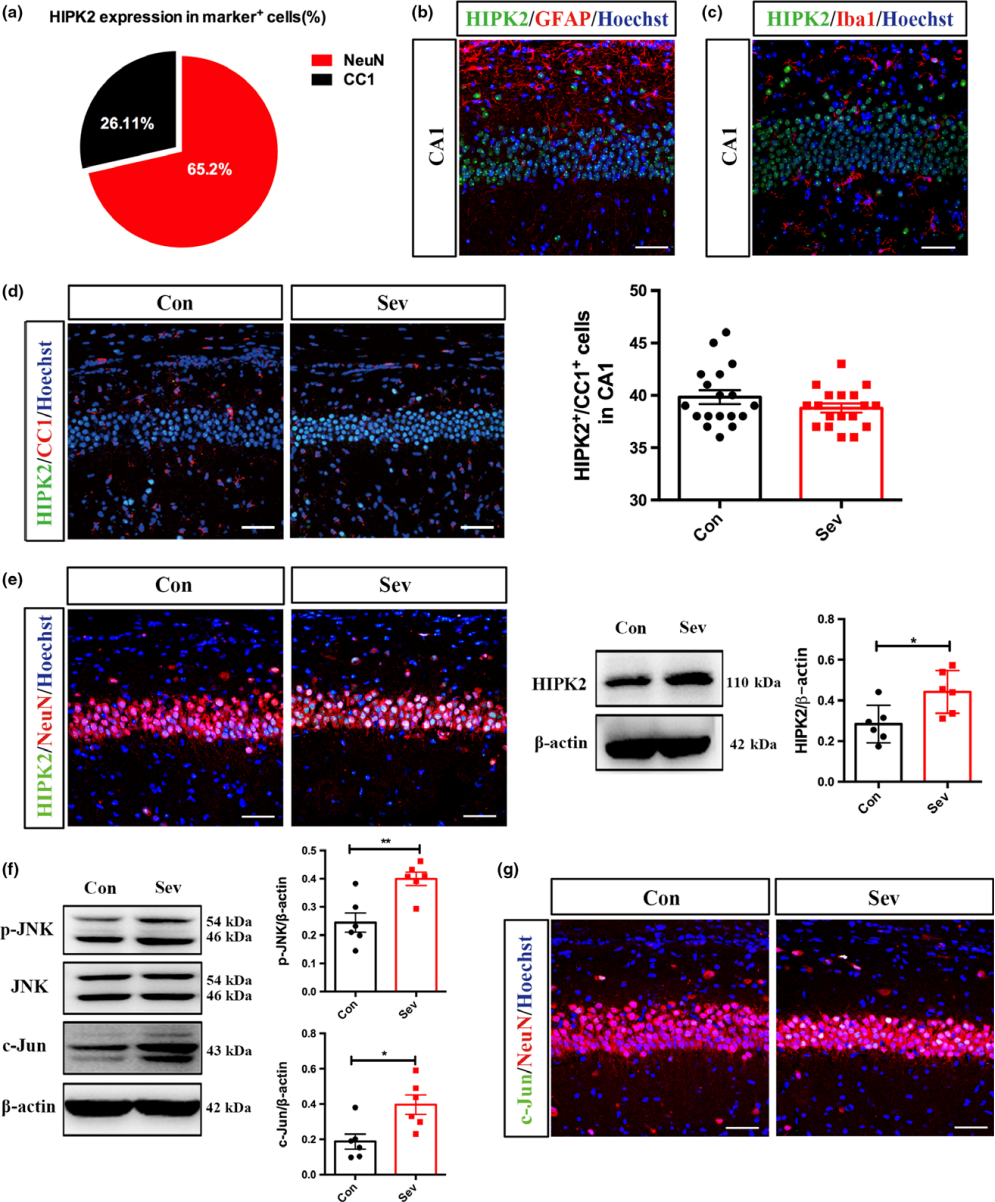
Effects of neonatal Sev exposure on the expression of homodomain interacting protein kinase 2 (HIPK2) and JNK/c‐Jun**.** (a) Quantification of HIPK2 expression in neural cell types. (b) Double‐immunostaining of HIPK2/GFAP in hippocampus. (c) Double‐immunostaining of HIPK2/Iba‐1 in hippocampus. (d) Double‐immunostaining of CC1/HIPK2 in the hippocampus of control rats and Sev‐treated rats. *N* = 6 rats per group. (e) Double‐immunostaining of NeuN/HIPK2 and western blotting of HIPK2 in the hippocampus of control rats and Sev‐treated rats at 6 h post last Sev treatment. Notice that HIPK2 is mainly expressed by neurons and Sev induces up‐regulation of HIPK2 in hippocampal neurons. *N* = 6 rats per group. (f) Western blotting of p‐JNK, JNK, and c‐Jun in the hippocampus of control and Sev‐treated rats at 6 h post last Sev treatment. *N* = 6 rats per group. (g) Double‐immunostaining of c‐Jun/NeuN in the hippocampus of control rats and Sev‐treated rats. Notice that up‐regulation of JNK/c‐Jun signaling upon Sev treatment. Con, control. Sev, Sevoflurane. *p < 0.05. **p < 0.01. Student’ *t*‐test (d–f). Bars = 50 μm.

**Figure 4:**
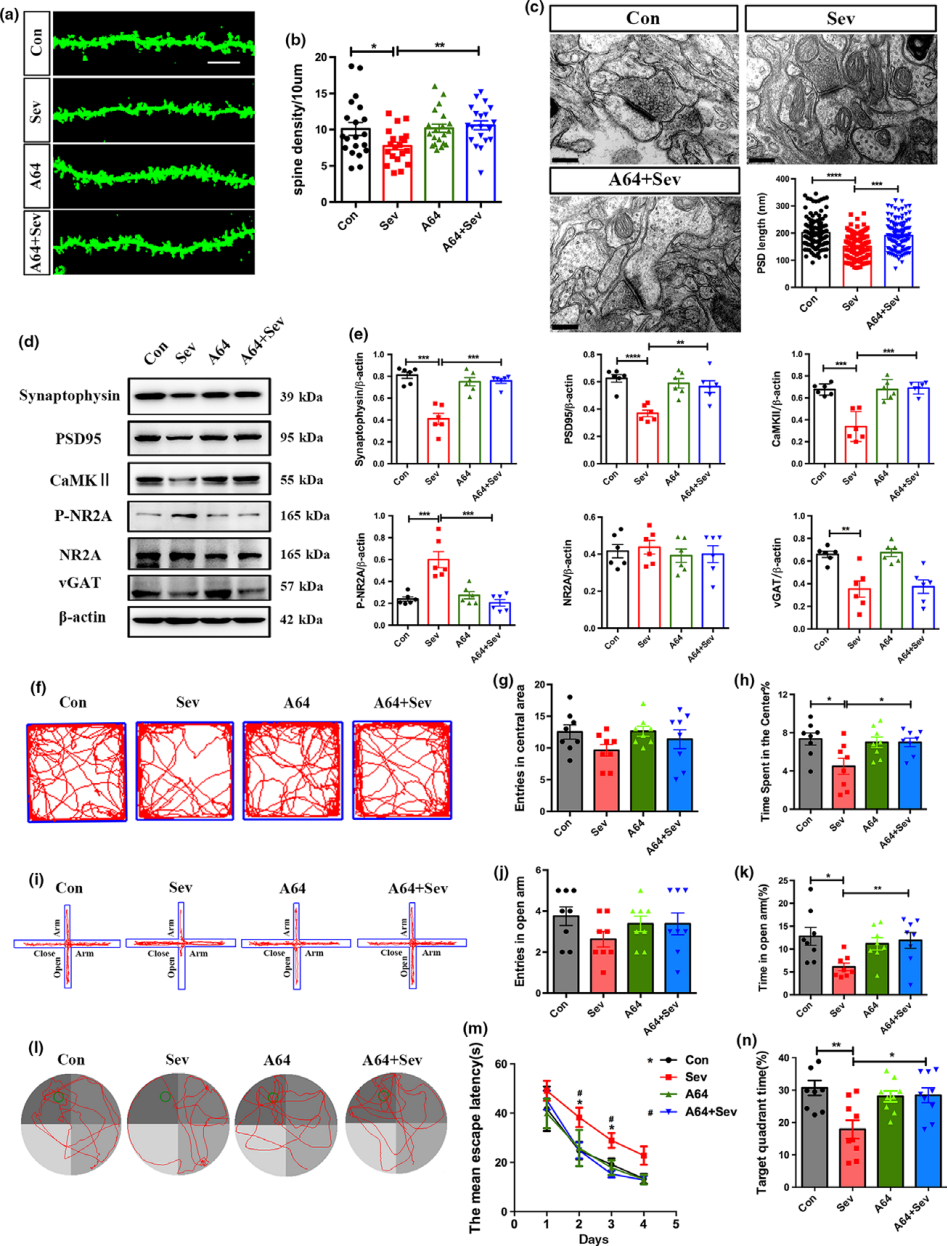
Effects of antagonizing homodomain interacting protein kinase 2 on the synaptic toxicity of Sev. (a, b) Golgi staining and quantification of spines in control rats, Sev‐treated, A64‐treated, and Sev plus A64‐treated rats. *N* = 6 rats per group. (c) Synaptic ultrastructure in hippocampus of control rats, A64‐treated, Sev‐treated, and Sev plus A64‐treated rats. Notice that the decrease in spine number and PSD length were partially rescued by A64. *N* = 6 rats per group. (d, e) Western blotting and quantification of Synaptophysin, post‐synaptic density protein 95 (PSD95), calcium/calmodulin‐dependent kinase II (CaMKII), phosphorylation of *N*‐methyl‐d‐aspartate receptor subunit 2 A (pNR2A), NR2A and vesicular inhibitory amino acid transporter (vGAT). Notice that the decrease in Synaptophysin, PSD95, CaMKII, and pNR2A were partially rescued by A64. *N* = 6 rats per group. (f–h) Open field assay. *N* = 8 rats per group. (i–k) Elevated plus maze assay. Notice that A64 treatment alleviated the Sev‐induced anxiety. *N* = 8 rats per group. (l–n) Morris water maze test. Notice that A64 treatment partially improved the spatial memory of Sev‐treated rats. *N* = 8 rats per group. Con, control. Sev, Sevoflurane. *p < 0.05. **p < 0.01. ***p < 0.001. ****p < 0.0001. ^#^ *p* < 0.05. One‐way anova (a–n). Bar = 10 μm (a) and 200 nm (c).

**Figure 5:**
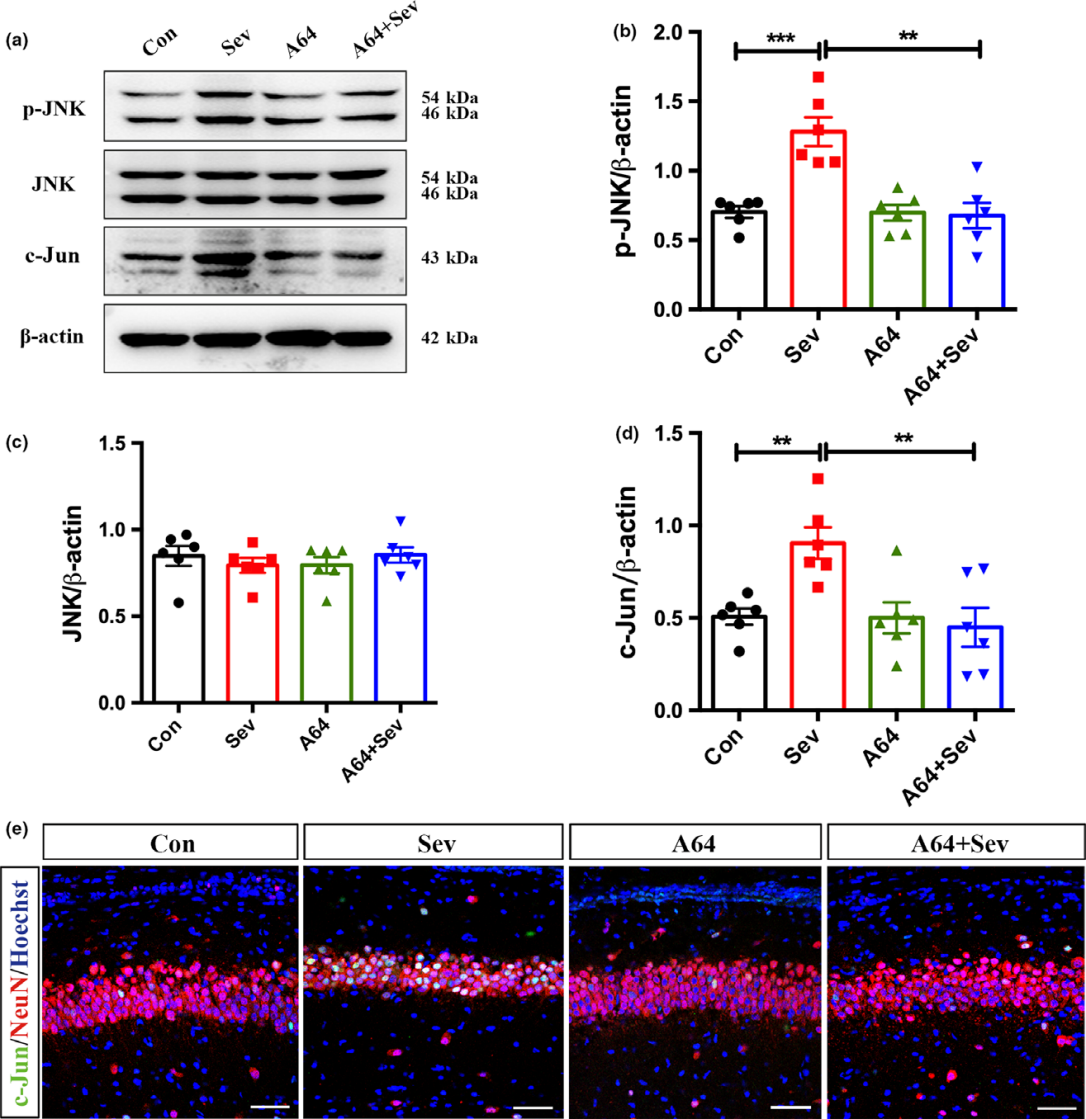
Effects of antagonizing homodomain interacting protein kinase 2 on the activation of JNK/c‐Jun signaling. (a–d) Western blotting and quantification of p‐JNK, JNK, c‐Jun in the hippocampus of control rats, rats treated with Sev, rats treated with A64, and rats treated with Sev plus A64. *N* = 6 rats per group. (e) Double‐immunostaining of c‐Jun/NeuN in the hippocampus of control rats, rats treated with Sev, rats treated with A64, and rats treated with Sev plus A64. Con, control. Sev, Sevoflurane. **p < 0.01. ***p < 0.001. One‐way anova (a–d). Bars = 50 μm.

**Figure 6:**
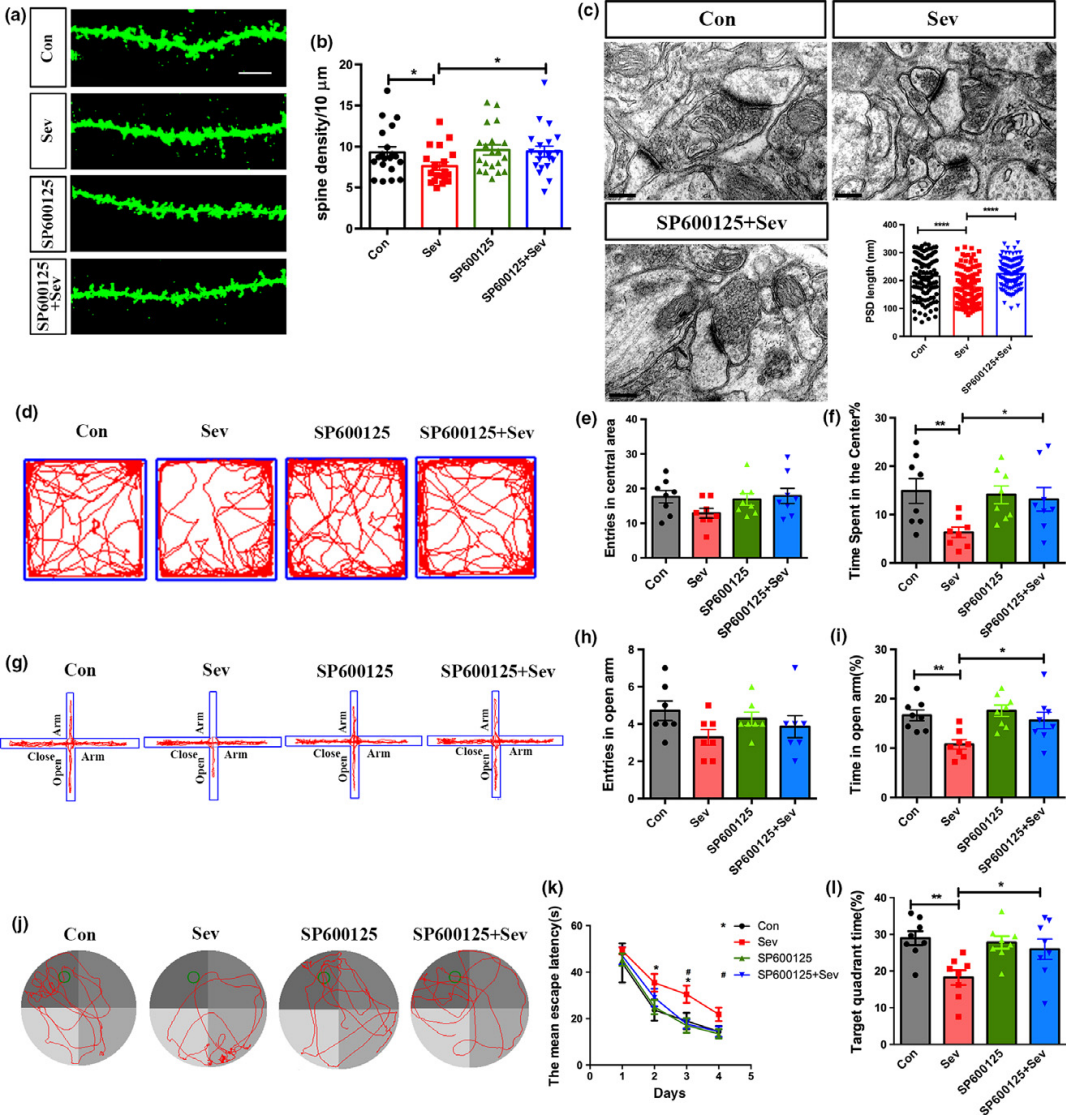
Effects of antagonizing JNK/c‐Jun on the synaptic toxicity of Sev. (a, b) Golgi staining and quantification of spines in control rats, Sev‐treated, SP600125‐treated, and Sev plus SP600125‐treated rats. *N* = 6 rats per group. (c) Synaptic ultrastructure in hippocampus of control rats, Sev‐treated, and Sev plus SP600125‐treated rats. Notice that the decrease in spine number and PSD length could be partially rescued by SP600125. *N* = 8 rats per group. (d–f) Open field assay. *N* = 8 rats per group. (g–i) Elevated plus maze assay. Notice that SP600125 significantly attenuated Sev‐induced anxiety. *N* = 8 rats per group. (j–l) Morris water maze test. *N* = 8 rats per group. Notice that SP600125 partially restored spatial memory in Sev‐treated rats. Con, control. Sev, Sevoflurane. *p < 0.05. **p < 0.01. ***p < 0.001. ****p < 0.0001. ^#^ *p* < 0.05 in (k). One‐way anova (a–l). Bar = 10 μm (a) and 200 nm (c).

All behavior analysis and statistics were performed by an investigator who was blinded to experimental design. No sample calculation was performed. At least eight rats were included in each group for behavior assay. For other experiments, at least six rats were included in each group for comparison. Each behavior test was conducted using distinct groups of animals. Only in cases when the mother rats did not breed Sev‐treated pups, those pups were excluded. Two pups were excluded and two additional pups from other litters were included. Data were presented as the mean ± standard error. All data were normally distributed as assessed by Shapiro–Wilk test (*p* values > 0.05). No test for outliers was conducted and no data were excluded Statistical comparisons were made using Student’s *t*‐test (Figs 1b–i,l, 2b,c,f,g, and 3d–f) or one‐way anova with Student–Newman–Keuls post hoc analysis (Figs 4, 5 and 6). *p* value less than 0.05 was considered as statistical significant.

## Results

### Effects of neonatal Sev exposure on the cognition function in adult

SD rats of 6 days old were exposed to 3% Sev for 2 h for three successive days. Spatial and fear memory, and anxiety behavior were analyzed when animals were 60–70 days old (Fig. 1a). Open field assay showed that Sev‐treated rats spent significantly much less time in the center of open field as compared to control rats (Fig. 1b–d). Elevated plus maze also revealed significant less time of Sev‐treated rats in the open arm (Fig. 1e–g), indicating the development of anxiety. In the conditioned fear memory test, fewer Sev‐treated rats exhibited freezing upon being placed in conditioned box (Fig. 1h and i). In Morris water maze assay which mainly assess the spatial memory, Sev‐treated rats spent significantly much more time for escaping and much less time exploring in the target quadrant as compared with control rats from 2 days post training (Fig. 1j–l). These data indicate that neonatal Sev treatment exerts long‐term harmful effects on the cognition function of rats.

### Effects of neonatal Sev exposure on neuron number and synapse development in hippocampus

To investigate the possible mechanism that underlies this long‐term toxic effect, we examined the effects of neonatal Sev exposure on apoptosis. Considering that Sev induced anxiety and showed harmful effects on memory, we examined the occurrence of apoptosis at 6 h post last Sev treatment in three key brain regions in regulating anxiety and memory which are hippocampus, anterior cingulate cortex (ACC), and caudate putamen (CPu). Interestingly, in ACC and CPu, most of the CC3‐positive cells were NeuN‐negative, although the levels of CC3 significantly increased in these two regions of Sev‐treated rats (Figure S1a–d). In hippocampus, significant increase in CC3 was detected in both the granular neurons and non‐neuron cells outside of granular layer (Fig. 2a and b). However, no significant changes in the total NeuN‐positive cells in hippocampal granular layer were found between control and Sev‐treated rats in adult (Fig. 2c). These data suggested that other factors may be involved in the dysfunction of hippocampus after Sev exposure.

Since hippocampus is involved in both memory and anxiety, we then examined the development of excitatory synapses in hippocampus at 8 weeks post‐Sev exposure. Golgi staining showed that the spine density along the dendrites of hippocampal granular neurons in Sev‐treated rats was significantly smaller than that in control rats (Fig. 2d). In regard of the spine morphology, no change in stubby spines was found between control and Sev‐treated rats. Both the number of mushroom‐shaped spines and thin spines decreased significantly in Sev‐treated rats (Fig. 2d), indicating the reduction of mature spines. At the ultrastructural level, the average length of post‐synapse density (PSD) was reduced by approximately 19% in Sev‐treated animals (Fig. 2e). Accordingly, a significant reduction in synaptophysin, PSD95, and CaMKII were observed in the hippocampus of rats treated by Sev (Fig. 2f and g). The phosphorylation of *N*‐methyl‐d‐aspartate receptor subunit 2 A (pNR2A) was significantly enhanced, whereas the expression of vGAT significantly decreased (Fig. 2f and g). These data indicate that neonatal Sev exposure imposes long‐term toxicity to synapse in hippocampus.

### Effects of neonatal Sev exposure on HIPK2‐JNK/c‐Jun signaling

To explore the intracellular signaling underpin the long‐term synaptic and behavior changes, we focused on the expression of HIPK2 and JNK/c‐Jun signaling in adult hippocampus, which has been reported to regulate the expression of NR2A (Shang *et al.* 2018). Double‐immunostaining of HIPK2 with NeuN (neuron marker), GFAP (astrocyte marker), CC1 (oligodendrocyte marker), and Iba‐1 (microglia marker) showed that HIPK2 was mainly expressed by neurons. Quantification showed that approximately 65.2% of HIPK2‐positive cells expressed NeuN and approximately 26.1 % of HIPK2‐positive cells were CC1‐positive (Fig. 3a). Very few astrocytes, or microglia expressed HIPK2 (Fig. 3b and c). After Sev treatment, there was no significant change in HIPK2/CC1‐positive cells (Fig. 3d).Importantly, up‐regulation of HIPK2 was observed mainly in neurons (Fig. 3e, left panels). Western blotting demonstrated that HIPK2 increased quickly after Sev‐treatment and kept high level till adult (Fig. 3e, right panels, and Figure S2a). These data indicated that Sev treatment up‐regulates the expression of HIPK2 in hippocampal granular neurons.

Similar as HIPK2, the phosphorylation of JNK in Sev‐treated rats increased significantly, whereas the total JNK remained unchanged (Fig. 3f). The levels of c‐Jun increased quickly with a similar time course as that of HIPK2 and kept high in adult as well (Fig. 3f and Figure S2b). Immunohistochemistry showed that c‐Jun was mainly induced in granular neurons (Fig. 3g). These data indicated that Sev treatment activates HIPK2‐JNK/c‐Jun signaling in hippocampal neurons.

### Antagonizing HIPK2 rescues Sev‐induced synapse impairment and blocks JNK/c‐Jun activation

To explore whether HIPK2 played a role in the Sev‐induced synapse toxicity, we applied a HIPK2 kinase inhibitor A64 (100 mg/kg) 1 h before each Sev exposure, and analyzed synaptic and behavior phenotype. Golgi staining showed that A64 treatment recovered the spine density to a similar level as control (Fig. 4a and b). At the ultrastructural level, the average length of PSD in rats treated with combination of Sev and A64 was significantly longer than that in rats treated with Sev alone (Fig. 4c). As to the synapse related proteins, A64 treatment significantly blocked the effects of Sev on the expression of synaptophysin, PSD95, CaMKII, and p‐NR2A, except for that of vGAT (Fig. 4d and e). In comparison with saline control, A64, by itself, did not affect the expression of synaptic proteins (Fig. 4d and e). These data indicated that inhibiting the kinase activity of HIPK2 could rescue the toxic effects of Sev on gluatmatic synapse development.

We next investigated the effects of A64 synaptic function of Sev‐treated rats. The Schaffer collateral pathway was stimulated at a threshold frequency (100 Hz, 1 s, four trains) that induced synaptic potentiation in all control rats, it lasted for more than 1 h with an amplitude increase of > 100% (Figure S3a). The potentiation of field post‐synaptic potentials (fPSPs) was significantly decreased in slices after Sevoflurane exposure. Pre‐incubation of A64 (100 mg/kg) 1 h before each Sev exposure partially reversed this effect (Figure S3b). Behavior assay showed that A64 treatment significantly enhanced the time spent in the center of the open field, and the time spent in the open arm of elevated plus maze (Fig. 4f–k). In water maze test, A64 treatment significantly reduced the escape latency and increased the frequency of target quadrant crossing (Fig. 4l–n). In fear conditioned memory assay, rats treated by A64 plus Sev were more prone to freezing as compared to rats treated by Sev alone (Figure S4).

We then investigated the effects of A64 on the activation of JNK/c‐Jun signaling. A64 treatment significantly attenuated the Sev‐induced JNK phosphorylation and c‐Jun up‐regulation (Fig. 5a–d).The number of NeuN/c‐Jun‐positive cells decreased significantly in the hippocampus of rats treated by Sev and A64 (Fig. 5e). Taken together, these data indicate that the kinase activity of HIPK2 is involved in Sev‐induced synaptic toxicity and JNK/c‐Jun activation.

### Effects of blocking JNK/c‐Jun signaling on the long‐term synaptic toxicity of Sev

We next assessed whether JNK/c‐Jun signaling was responsible for Sev’s synaptic toxicity. A specific JNK/c‐Jun inhibitor SP600125 (10 mg/kg) was administered in together with Sev treatment, and the number of spines was quantified at 8 weeks old. Rats which were treated by combination of Sev and SP600125 showed significantly more spines than rats treated by Sev alone (Fig. 6a and b). At the ultrastructural level, longer PSD was detected in rats treated by combination of SP600125 and Sev in comparison with rats treated by Sev, suggesting that blocking JNK/c‐Jun signaling could rescue the synapse impairment by Sev (Fig. 6c). SP600125, by itself, exerted no significant effects on spine density and PSD length.

In open field assay, rats treated with SP600125 plus Sev spent significantly much time in the center, even to a similar level of the normal control rats (Fig. 6d–f). In elevated plus maze test, rats treated with SP600125 plus Sev spent significantly much time in the open arm than rats treated with Sev alone (Fig. 6g–i). In Morris water maze test, SP600125 group showed significantly shortened the escape latency and longer time spent in the target quadrant, than rats treated by Sev (Fig. 6j–l). In fear conditioned memory assay, SP600125 significantly increased the percentages of rats exhibiting freezing (Figure S5a). Interestingly, administration of SP600125 did not affect the expression of HIPK2 (Figure S5b). These data indicated that JNK/c‐Jun signaling may be the key signaling downstream of HIPK2 in the Sev‐induced synapse toxicity.

## Discussion

In this study, we investigated the long‐term effects of neonatal repeated Sev exposure on the memory and anxiety behavior, and synapse development in adult. By analyzing the expression of HIPK2/JNK‐c‐Jun, and by pharmacological manipulation, we probed the possible underlying mechanism underlying Sev’s synaptic toxicity. The overall data supported a long‐term synaptic toxicity of neonatal Sev exposure and an involvement of HIPK2/JNK‐c‐Jun signaling in this process.

Previous studies have reported occurrence of apoptosis after repeated neonatal Sev exposure (Chen *et al.* 2016; Zhou *et al.* 2016a; Zhou *et al.* 2016b). Mechanistically, endoplasmic reticulum stress (Chen *et al.* 2013; Liu *et al.* 2017; Zhu *et al.* 2017), autophagy (Xiao *et al.* 2017; Li *et al.* 2017; Xu *et al.* 2018a; Xu *et al.* 2018b), and microRNA (such as microRNA188, microRNA96) have been indicated to account for the apoptosis‐inducing effect of Sev (Zhou *et al.* 2017; Wang *et al.* 2018; Xu *et al.* 2019). Surprisingly, our data showed that Sev‐induced apoptosis were mainly glial cells in ACC and CPu. Although neuronal apoptosis was detected in the granular layer of hippocampus, the number of granular neurons remained unchanged in adult, indicating that neuronal apoptosis may not be the major cause of cognition impairment. It is known that there was continuous neurogenesis in adult hippocampus. Previous studies have reported that Sev‐treatment could lead to acute loss of hippocampal neurons, and hippocampal neural stem cells respond actively to injury. It is possible that compensate neurogenesis may recovered the number of granular neurons in Sev‐treated rats, which is of interesting to be further explored.

As a widely used anesthetic and sedative, the mechanism that Sev renders animals painless and unconsciousness has been thought to be mediated mainly by GABA receptors (Ogawa *et al.* 2011; Liu *et al.* 2016; Brohan and Goudra 2017). Recently, researchers noticed that Sev treatment might also affect glutamate release (Moe *et al.* 2002) and glutamatergic neurotransmission (Stucke *et al.* 2005). Our data demonstrated that neonatal Sev‐exposure led to significant decrease in spine number and glutamate receptors in adult hippocampus, strongly suggesting a long‐term toxic effect of Sev on excitatory synapse, which may contribute to the impairment of memory and appearance of anxiety.

To explore the underlying mechanism, we focused on HIPK2‐JNK/c‐Jun signaling. A recent study has identified HIPK2‐JNK‐c‐Jun signaling as a key mechanism that regulates the transcription of NMDA receptor subunits NR2A and NR2C (Shang *et al.* 2018), suggesting that HIPK2‐JNK‐c‐Jun signaling may be involved in the synaptic plasticity. Previous studies have demonstrated that nuclear activation of JNK‐c‐Jun signaling regulates the expression of glutamate receptors through nuclear‐synapse communication (Kravchick *et al.* 2016) and synaptic JNK‐c‐Jun signaling regulates glutamate release (Nistico *et al.* 2015). The rapid response of HIPK2 and the effectiveness of A64 indicated a crucial role of HIPK2 in the synaptic toxicity of Sev. Interestingly, A64 treatment rescued the effects of Sev on the phosphorylation of NR2A, but not the expression of vGAT, indicating that HIPK2 may function mainly in glutamatergic neurons. The facts that A64 affected JNK/c‐Jun, whereas SP600125 did not affect the expression of HIPK2 suggested that JNK/c‐Jun may be the downstream signaling of HIPK2. A caveat of our study is not providing evidence on whether JNK/c‐Jun is directly phosphorylated by HIPK2. Although we do not exclude the involvement of other downstream substrates of HIPK2 in this process, the effects of SP600125 indicated that JNK/c‐Jun signaling partially mediates the synaptic toxicity of Sev. In together, our data revealed an important role of HIPK2/JNK/c‐Jun signaling in the long‐term synaptic toxicity of neonatal Sev exposure.

### Author contributions

Behavior analysis: L.L. L.R., L.B.; Golgi staining: L.L. L.R.; Immunohistochemistry: L.L. L.R. W.X.; Electron microscopic study: L.L., W.F.; Western blotting: L.L. L.R., M.R.; Patch‐clamp recording: X. R; Data analysis: L.L. Z.H., L.R.; Manuscript preparation: L.L., Z.H., W.Y.; Experimental design: L.L., Z.H., W.Y. W.S.; Financial support: Z.H.

### OPEN SCIENCE BADGES

This article has received a badge for *Open Materials* because it provided all relevant information to reproduce the study in the manuscript. More information about the Open Practices badges can be found at https://cos.io/our-services/open-science-badges/.

## Supporting information


**Figure S1**. Expression of CC3 in ACC and CPu.
**Figure S2**. Western blotting of HIPK2 in control and Sev‐treated rats in adult (a). Western blotting of p‐JNK, JNK, and c‐Jun in control and Sev‐treated rats in adult (b, c).
**Figure S3**. Extracellular recording of field post‐synaptic potentials (fPSPs) in control, Sev‐treated, Sev + A64 treated rats. Notice that Sev suppressed the long‐term potential and A64 partially rescued this effect.
**Figure S4**. Fear conditioned memory assay in control, Sev‐treated, A64 treated, and Sev + A64 treated rats. Notice that A64 treatment could rescue Sev induced memory impairment.
**Figure S5**. Effects of SP600125 on the fear memory and expression of HIPK2.
