# Supplementary material for: Involvement of homodomain interacting protein kinase 2‐c‐Jun N‐terminal kinase/c‐Jun cascade in the long‐term synaptic toxicity and cognition impairment induced by neonatal Sevoflurane exposure
Source: J Neurochem. 2020 Jan 19;154(4):372–88. doi: 10.1111/jnc.14910 (PMC7496229; doi:10.1111/jnc.14910)
Supplement: Supplementary file 1 — Figure S1. Expression of CC3 in ACC and CPu. Figure S2. Western blotting of HIPK2 in control and Sev‐treated rats in adult (a). Western blotting of p‐JNK, JNK, and c‐Jun in control and Sev‐treated rats in adult (b, c). Figure S3. Extracellular recording of field post‐synaptic potentials (fPSPs) in control, Sev‐treated, Sev + A64 treated rats. Notice that Sev suppressed the long‐term potential and A64 partially rescued this effect. Figure S4. Fear conditioned memory assay in control, Sev‐treated, A64 treated, and Sev + A64 treated rats. Notice that A64 treatment could rescue Sev induced memory impairment. Figure S5. Effects of SP600125 on the fear memory and expression of HIPK2. [file JNC-154-372-s001.pdf]

Supplementary information

Title: Involvement of HIPK2-JNK/c-Jun cascade in the long term synaptic toxicity and cognition impairment induced by neonatal Sevoflurane exposure

Authors: Lirong Liang[1#], Rougang Xie[1#], Rui Lu[1#], Ruixue Ma[1], Xiaoxia Wang[1], Fengjuan Wang[1], Bing Liu[1], Shengxi Wu[2], Yazhou Wang[2]*, Hui Zhang[1]*

Supplementary figure legends

Supplementary Figure 1. Expression of CC3 in ACC and CPu. (A) Double-immunostaining of CC3/NeuN in the ACC of control and Sev-treated rats. (B) Western-blotting of CC3 in the ACC of control and Sev-treated rats. (C) Double-immunostaining of CC3/NeuN in the CPu of control and Sev-treated rats. (D) Western-blotting of CC3 in the CPu of control and Sev-treated rats. Notice the expression of CC3 in non-neuron cells. Con, control. Sev, Sevoflurane. N = 6 rats per group. *$P<0.05$. Student's *t* test. Bars = 50μm.

Supplementary Figure 2. Western-blotting of HIPK2 in control and Sev-treated rats in adult (A). Western-blotting of p-JNK, JNK and c-Jun in control and Sev-treated rats in adult (B,C). Con, control. Sev, Sevoflurane. N = 6 rats per group. *$P<0.05$. **$P<0.01$. Student's t test.

Supplementary Figure 3. Extracellular recording of field postsynaptic potentials (fPSPs) in control, Sev-treated, Sev+A64 treated rats. Notice that Sev suppressed the long term potential and A64 partially rescued this effect. N = 5-6 neurons from 2-3 rats per group. *P<0.05. One-way ANOVA.

Supplementary Figure 4. Fear conditioned memory assay in control, Sev-treated, A64 treated, and Sev+A64 treated rats. Notice that A64 treatment could rescue Sev induced memory impairment. Con, control. Sev, Sevoflurane. N = 6 rats per group. *P<0.05. One-way ANOVA.

Supplementary Figure 5. Effects of SP600125 on the fear memory and expression of HIPK2. (A) Fear conditioned memory assay in control, Sev-treated, Sev+SP600125 treated rats. Notice that SP600125 treatment could partially rescue Sev induced memory impairment. (B) Western-blotting of HIPK2 in control, Sev-treated, Sev+SP600125 treated rats. Con, control. Sev, Sevoflurane. N = 6 rats per group.*P<0.05. One-way ANOVA.

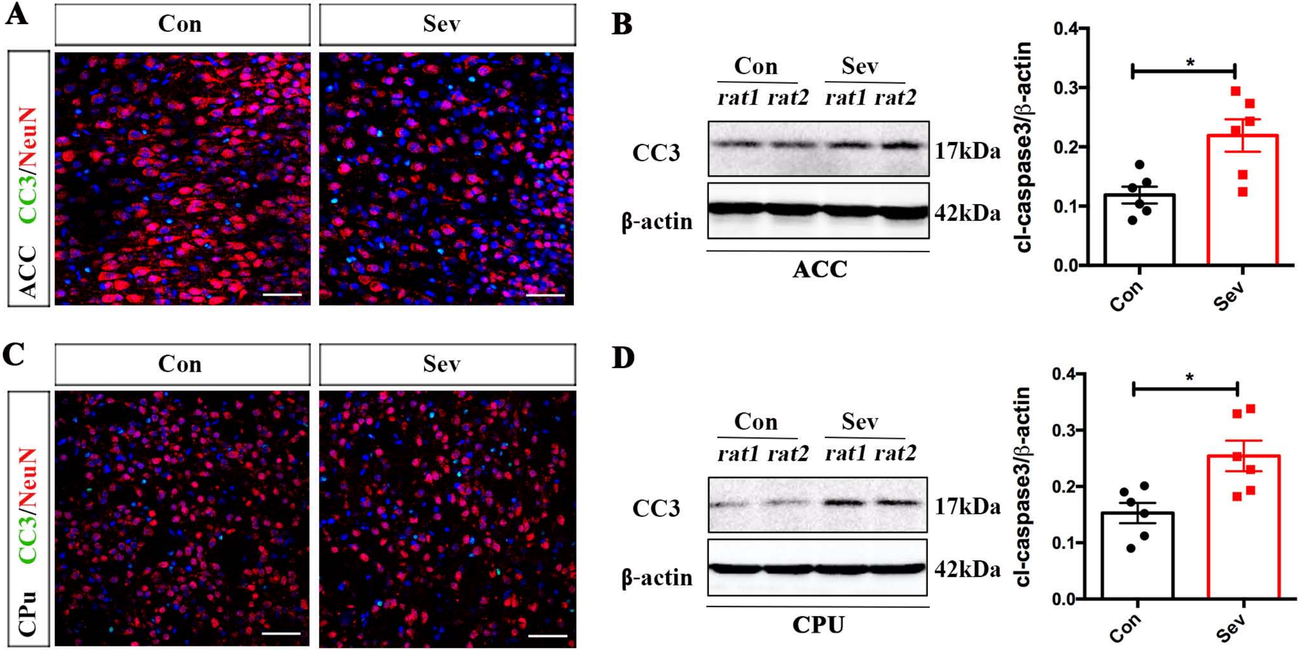

Supplementary Fig-1.

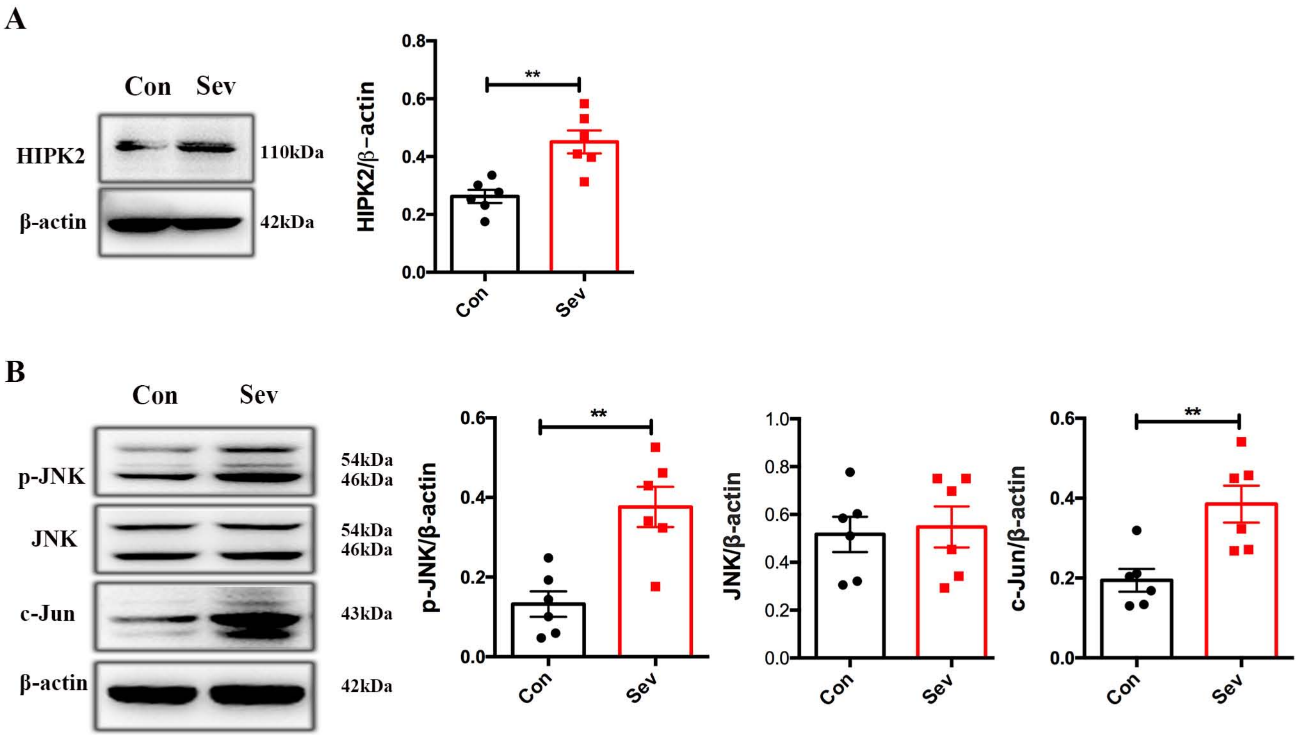

Supplementary Fig-2

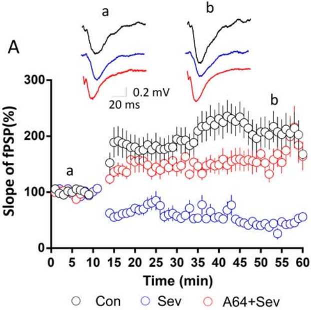

Supplementary Fig-3

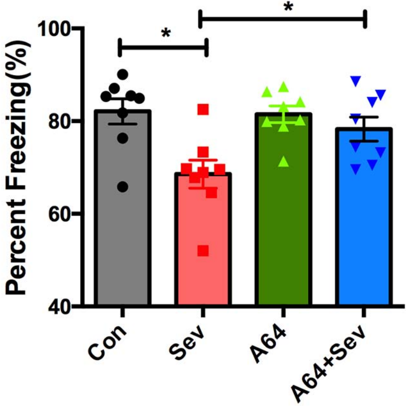

Supplementary Fig-4

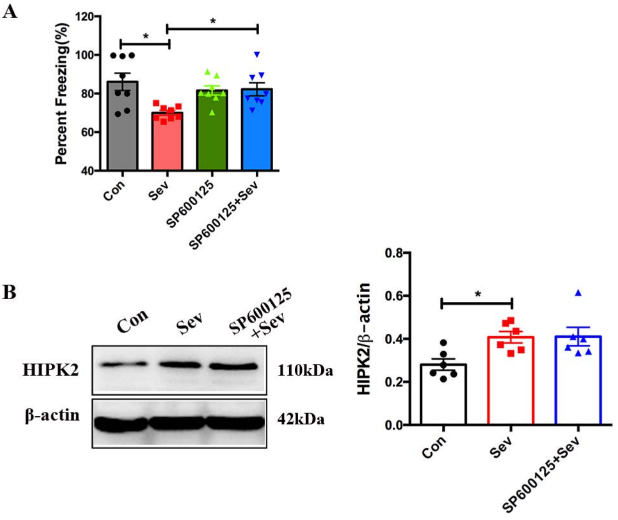

Supplementary Fig-5
